# Analysis toolkit for evaluation of drug titration practice in acute lymphoblastic leukemia maintenance

**DOI:** 10.1093/jamiaopen/ooae089

**Published:** 2024-09-13

**Authors:** Tushar Mungle, Ananya Mahadevan, Jayanta Mukhopadhyay, Sangeeta Das Bhattacharya, Vaskar Saha, Shekhar Krishnan

**Affiliations:** School of Medical Sciences and Technology, Indian Institute of Technology Kharagpur, Kharagpur, West Bengal, 721 302, India; Tata Translational Cancer Research Centre, Tata Medical Center, Kolkata, West Bengal, 700 160, India; Tata Translational Cancer Research Centre, Tata Medical Center, Kolkata, West Bengal, 700 160, India; Department of Computer Science and Engineering, Indian Institute of Technology Kharagpur, Kharagpur, West Bengal, 721 302, India; School of Medical Sciences and Technology, Indian Institute of Technology Kharagpur, Kharagpur, West Bengal, 721 302, India; Tata Translational Cancer Research Centre, Tata Medical Center, Kolkata, West Bengal, 700 160, India; Department of Paediatric Haematology and Oncology, Tata Medical Center, Kolkata, West Bengal, 700 160, India; Division of Cancer Sciences, School of Medical Sciences, Faculty of Biology, Medicine and Health, University of Manchester, Manchester, M13 9PL, United Kingdom; Tata Translational Cancer Research Centre, Tata Medical Center, Kolkata, West Bengal, 700 160, India; Department of Paediatric Haematology and Oncology, Tata Medical Center, Kolkata, West Bengal, 700 160, India; Division of Cancer Sciences, School of Medical Sciences, Faculty of Biology, Medicine and Health, University of Manchester, Manchester, M13 9PL, United Kingdom

**Keywords:** acute lymphoblastic leukemia, maintenance therapy, drug titration, visualization and analysis, protocol compliance

## Abstract

**Objective:**

During the 2-year maintenance treatment phase (MT) of acute lymphoblastic leukemia (ALL), personalized patient-specified titration of oral antimetabolite drug doses is required to ensure maximum tolerated systemic drug exposure. Drug titration is difficult to implement in practice and insufficient systemic drug exposure resulting from inadequate dose titration increases risk of ALL relapse.

**Materials and Methods:**

We developed an open-source R-based analytical toolkit, including the allMT R package and an interactive web-based R Shiny VIATAMIN application, to evaluate antimetabolite drug titration during MT.

**Results:**

Evaluation is initiated with basic visualization analysis of drug titration, in both individual patients and patient cohorts. Observations are supplemented with descriptive analyses of hematological toxicity frequency and prescriber compliance rates with protocol drug titration rules. In individual patients, visual and quantitative analyses indicate recurring potentially correctable suboptimal drug titration patterns. In patient cohorts, the toolkit enables evaluation of the impact of interventions to optimize MT drug titration.

**Discussion:**

Incorporation of the toolkit in a forthcoming clinical decision support system for MT would enable real-time examination and course correction of drug titration practice.

**Conclusion:**

Future versions will be enhanced to include other variables that influence drug titration practice, including clinical toxicity data and later, pharmacological markers of antimetabolite, adherence, and drug activity.

## Introduction

With contemporary treatment, cure rates of pediatric acute lymphoblastic leukemia (ALL) approach 90%.^1^ Treatment spans 2.5-3.5 years and includes an intensive phase followed by a 2-to-3-year maintenance phase. Treatment during the intensive phase is determined by risk-adapted therapy, which establishes the combination, schedule, and dose of chemotherapy drugs. Unlike the intensive phase, ALL maintenance treatment (ALL·MT) is generally similar in all risk groups and entails treatment with the oral antimetabolite drugs 6-mercaptopurine (6MP) and methotrexate (MTX). A cornerstone of ALL·MT is to ensure sufficient and continuous systemic exposure to the antimetabolite drugs for the duration of treatment.^2^^,^^3^ Observations from pharmacological monitoring of intracellular and DNA-bound active drug metabolites support this principle.^2^^,^^4^ In clinical practice, this necessitates continuous adjustments to antimetabolite dosages throughout ALL·MT. This process of drug titration, tailored to each patient, is fundamental to ALL·MT therapy.^5^^,^^6^ Drug titration involves systematically modifying antimetabolite doses, upward and downward, to achieve and maintain the maximum tolerated drug combination for each patient. Drug titration is individualized to account for patient-specific differences in drug metabolism and organ function that influence susceptibility to drug-related toxicity. In the absence of routine pharmacological monitoring, antimetabolite dose titrations in ALL·MT are guided by target blood counts, particularly the neutrophil count.

Although a fundamental treatment principle, there are currently no readily evaluable measures of drug titration practice in ALL·MT. In this report, we describe the development of simple visual representations of drug titration practice in ALL·MT. These visualizations are based on routinely used clinical parameters and are designed for monitoring drug titration practice in ALL·MT, both for individual patients and patient cohorts. Interpretation of these visualizations is facilitated using quantitative assessments of treatment-related (hematological) toxicity and prescriber compliance to drug titration rules. The proposed instrument thus enables evaluation of the implementation of drug titration in ALL·MT, adherence to predefined guidelines, and the influence of drug-related toxicity and/or prescriber non-adherence on the observed deviations. We have provided these assessment measures as an open-access R-based analysis toolkit, available both as an R package (allMT R library)^7^ and as an interactive user-friendly web-based R/Shiny application (VIATAMIN, Visualisation & Analysis Tool in ALL Maintenance).^8^ The proposed analytical toolkit may be employed both as a quality improvement initiative to improve drug titration practice and as an audit instrument to evaluate practice and identify opportunities for improvement.

## Methods

### Patients

Patients received maintenance treatment as part of an Institutional Review Board-approved Indian Collaborative Childhood Leukaemia protocol^9^ for the treatment of patients aged 1-18 years with newly diagnosed ALL at Tata Medical Center Kolkata (ICiCLe-ALL-14; IRB approval EC/TMC/12/13). All data were collected and analyzed within the scope of this IRB-approved study. Study patients began maintenance treatment between March 13, 2014, and March 24, 2022, and their anonymized data files (including serial blood counts and antimetabolite drug doses) are available in the Mendeley Data repository.^10^Tables S1 and S2 detail typical drug titration rules and data capture methods, respectively. Further details are available in the Supplementary Material.

### Analysis methods to evaluate drug titration practice in ALL·MT

**Figure 1.**
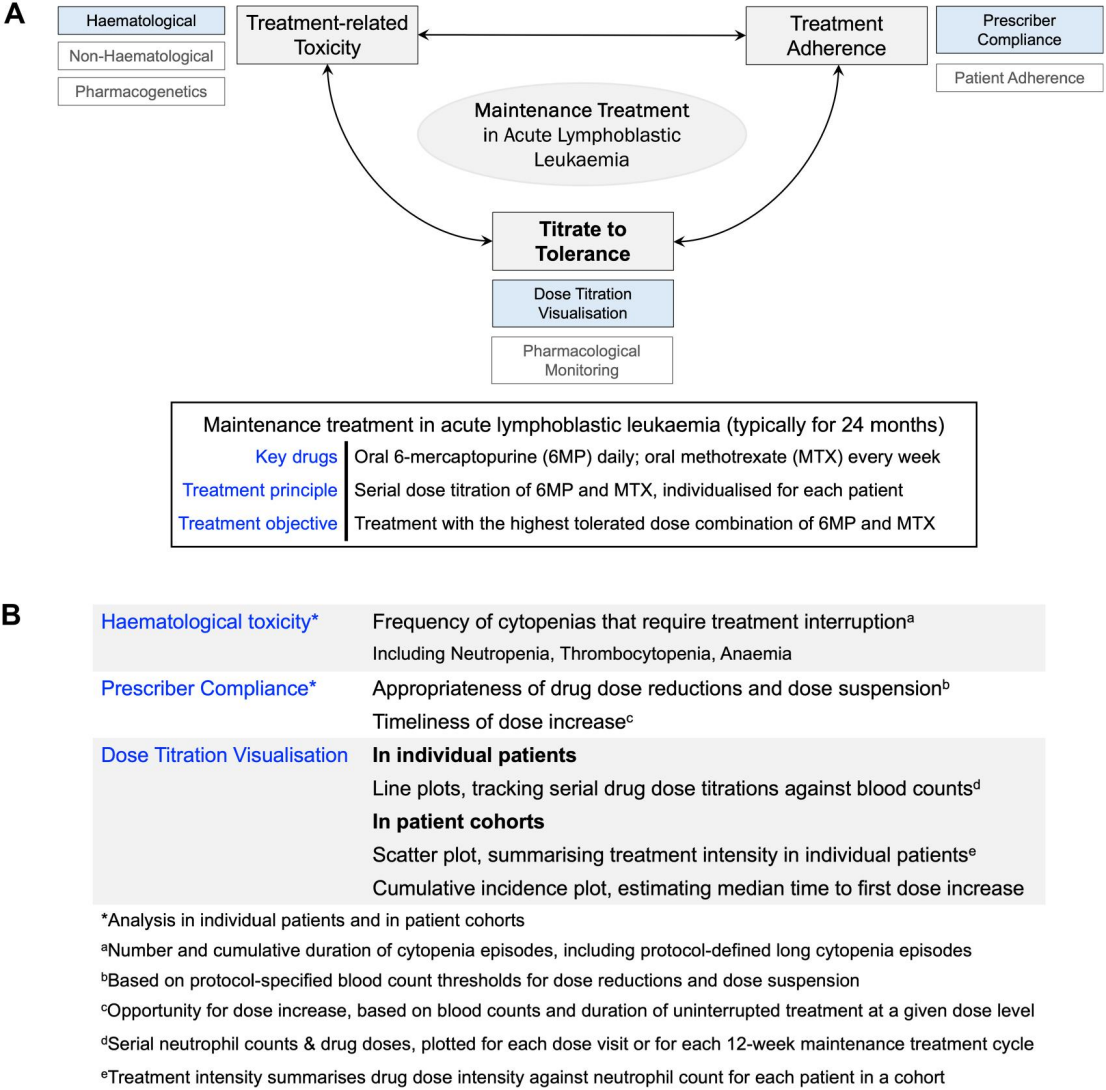
Auditing dose titration practice during the maintenance treatment phase in acute lymphoblastic leukemia (ALL). (A) The central treatment tenet during the ALL-maintenance treatment phase (characteristically 24 months long) is to administer the highest tolerated dose combination of the oral antimetabolite drugs 6-mercaptopurine (6MP) and methotrexate (MTX). This requires serial drug dose titrations, individualized for each patient (the “Titrate to Tolerance” principle). Dose titration is influenced both by treatment-related toxicity and adherence to treatment. The proposed analysis toolkit (filled text boxes) employs visualizations to evaluate dose titration practice, in conjunction with summary statistics to examine the impact of prescriber compliance (with protocol-specified dose titration rules) and hematological toxicities on dose titration practice, both in individual patients and in patient cohorts. Future versions of the audit toolkit (outlined in lighter font) would include characterization of recurring non-hematological toxicities (eg, viral infections, toxicity related to methylated 6MP metabolites), pharmacogenetic determinants (eg, germline variants influencing drug metabolism), and pharmacological monitoring (to estimate systemic drug exposure and to monitor patient adherence). (B) Outline of component elements of each analysis objective (hematological toxicity, prescriber compliance, and visualization of dose titration practice) in individual patients and in patient cohorts.

Analysis methods include visual representations to examine the implementation of antimetabolite drug titration and quantitative measures to assess factors influencing titration practice. Dose titration visualization in individual patients employs line plots to track antimetabolite drug doses and blood counts, both for each prescribing visit (ie, antimetabolite doses prescribed in mg/week and the corresponding neutrophil count at each visit) and for each 12-week maintenance treatment cycle (ie, weighted means of neutrophil count and antimetabolite dose intensity for each of the eight 12-week maintenance treatment cycles). In patient cohorts, scatter plots depict the weighted means of neutrophil count and antimetabolite dose intensity over the entire course of ALL·MT for individual patients in the cohort. Aggregate antimetabolite dose exposure and associated blood count values between prescribing visits are represented using weighted means. Antimetabolite dose exposure is determined by multiplying the weighted mean dose intensities of 6-mercaptopurine and methotrexate. Dose intensity is calculated as the ratio of the prescribed drug dose to the recommended protocol dose. Quantitative measures include descriptive data of treatment-related hematological toxicity (including frequency and cumulative duration of neutropenia, anemia, and thrombocytopenia episodes necessitating treatment suspension) and the extent of prescriber compliance with protocol rules for dose modifications (including dose reduction, treatment suspension, and increase in antimetabolite doses). Figure 1 highlights the interaction between drug titration practice, treatment adherence, and treatment-related toxicity and outlines the analysis measures used to represent each of these factors.

Table 1 summarizes the visualization and assessment functions that comprise the titration analysis toolkit developed using the R program, available both as a standalone R package (allMT) and as base for an interactive web-based R Shiny application (VIATAMIN).

**Table 1.** The collection of R functions in the allMT R package, and the accompanying web-based interactive VIATAMIN R Shiny application, for audit of antimetabolite dose titration practice and treatment-related hematological toxicity in individual patients and in patient cohorts, during the maintenance treatment phase of ALL.

		Output	Analysis objective	allMT R Package	VIATAMIN R Shiny app
	**Data upload^a^**				
	Data transformation			Functions 1, 2	Not included
	**Analysis in individual patients**				
	Tracking serial blood counts and drug doses across MT	Graph	Dose titration	Function 3	Yes
	Tracking treatment intensity serially across MT cycles	Graph	Dose titration	Function 4	Yes
	Time to first dose escalation	Value	Dose titration	Function 5	Yes
	Neutropenia: frequency and cumulative duration (in weeks)	Table	Hematological toxicity	Function 6	Yes
	Thrombocytopenia: frequency and cumulative duration (in weeks)	Table	Hematological toxicity	Function 7	Yes
	Anemia: frequency and cumulative duration (in weeks)	Table	Hematological toxicity	Function 8	Yes
	**Analysis of patient cohorts**				
	Treatment intensity scatter plot	Graph	Dose titration practice	Function 9	Yes
	Comparison of treatment intensity between cohorts	Graph	Dose titration practice	Function 10	Yes
	Median time to first dose escalation	Graph	Dose titration practice	Function 5	Yes
	Neutropenia: frequency and cumulative duration (in weeks)	Table	Hematological toxicity	Function 6	Yes
	Thrombocytopenia: frequency and cumulative duration (in weeks)	Table	Hematological toxicity	Function 7	Yes
	Anemia: frequency and cumulative duration (in weeks)	Table	Hematological toxicity	Function 8	Yes
	**Analysis of compliance with dose titration rules [individual and patient cohorts]**				
	Compliance with “STOP DOSE” rule	Table	Practitioner compliance	Function 11	Yes
	Compliance with “REDUCE DOSE” rule	Table	Practitioner compliance	Function 12	Yes
	Compliance with “INCREASE DOSE” rule	Table	Practitioner compliance	Function 13	Yes
1	convert_tmc_format() [*Usage*: “convert_tmc_format(inputpath_to_excelfolder, exportpath_to_csvfolder, daily_mp_dose, weekly_mtx_dose”]				
2	convert_external_format() [*Usage*: “convert_external_format(inputpath_to_excelfolder, exportpath_to_csvfolder, pat_data_file_path, daily_mp_dose, weekly_mtx_dose”]				
3	plot_progression() [*Usage*: “plot_progression(input_file_path, anc_range, unit)”]				
4	summarize_cycle_progression() [*Usage*: “summarize_cycle_progression(input_file_path, anc_range, unit)”]				
5	time_to_first_dose_increase() [*Usage*: “time_to_first_dose_increase(input_files_path, escalation_factor)”]				
6	assess_neutropenia() [*Usage*: “assess_neutropenia(input_files_path, anc_range, duration_anc = NA)”]				
7	assess_thrombocytopenia() [*Usage*: “assess_thrombocytopenia(input_files_path, duration_plt = NA)”]				
8	assess_anemia() [*Usage*: “assess_anemia(input_files_path, hb_range, duration_hb = NA)”]				
9	summarize_cohortMT() [*Usage*: “summarize_cohortMT(input_files_path, anc_range, unit, dose_intensity_threshold)”]				
10	compare_cohorts() [*Usage*: “compare_cohorts(input_files_path, unit, anc_range, dose_intensity_threshold, method, intervention_date, group_data_path)”]				
11	assess_stop_doses() [*Usage*: “assess_stop_doses(input_files_path, anc_threshold = NA, plt_threshold = NA, hb_threshold = NA)”]				
12	assess_reduced_doses() [*Usage*: “assess_reduced_doses(input_files_path, anc_range = NA, plt_range = NA, hb_range = NA, reduction_factor)”]				
13	assess_increased_doses() [*Usage*: “assess_increased_doses(input_files_path, anc_threshold = NA, plt_threshold = NA, hb_threshold = NA, escalation_factor, tolerated_dose_duration)”]				

allMT Library source code: Source code: <https://github.com/tmungle/allMT>; User guide: https://tmungle.github.io/allMT/reference/index.html; VIATAMIN: Visualisation & Analysis Tool in ALL Maintenance R Shiny app (https://ananyam.shinyapps.io/VIATAMIN/).

ALL, acute lymphoblastic leukemia; ANC, absolute neutrophil count; Hb, hemoglobin; MT, maintenance treatment; PLT, platelet count.

a Template data recording format may be downloaded after installing the allMT R package using the following commands:

pat_data <- system.file(“extdata/tmc_data/”, “UPN_916.xls”, package = “allMT”)

dest_path <- getwd()

file.copy(pat_data, dest_path)

## Results

### The VIATAMIN R Shiny application for visualization and analysis of drug titration practice in ALL·MT

VIATAMIN^8^ is an interactive user-friendly open-source R-based web application for the analysis of drug titration practice during ALL maintenance, built using the allMT R package. The application features visualization tools to identify drug titration patterns, including dose up-titration, down-titration, non-titration, and related combinations. To understand the factors influencing the observed titration patterns, the application quantifies treatment-related toxicity and evaluates prescriber adherence to protocol-specified drug titration rules, such as stopping, reducing, or escalating drug doses. Treatment-related toxicity is assessed using blood count data (hemoglobin levels and absolute neutrophil and platelet counts) to pinpoint instances of low blood counts that warrant treatment modifications. These analyses can be conducted on individual patients or patient cohorts. Subsequent sections explore how VIATAMIN reveals recurring drug titration patterns and their potential causes.

### Analysis in individual patients highlights adherence to drug titration principles and reveals practice variations

**Figure 2.**
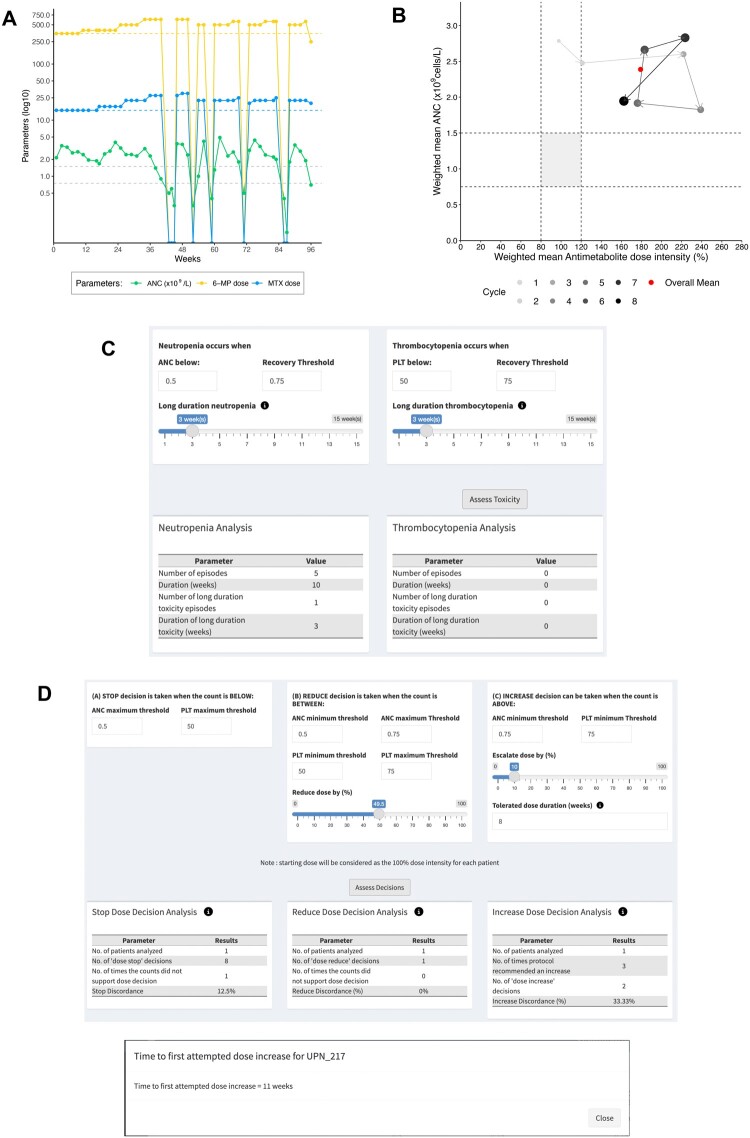
Illustration of the optimal “drug titration to tolerance” practice in ALL Maintenance*. Output from analysis of drug titration practice in an individual patient using the VIATAMIN R Shiny web application (https://ananyam.shinyapps.io/VIATAMIN/). Details are discussed in the text. (A) Longitudinal plot of serial neutrophil counts (×10^9^/L) and weekly oral antimetabolite drug doses (6-mercaptopurine and methotrexate; mg/week) prescribed at each dosing visit through the 96 weeks of ALL maintenance treatment. Horizontal dash lines indicate the protocol-specific recommended absolute neutrophil count range (ANC, 0.75-1.5 × 10^9^/L) and the protocol-recommended doses of 6-mercaptopurine (MP) and methotrexate (MTX) during maintenance treatment. (B) Cycle-specific summary measures plotting changes in ALL maintenance treatment intensity (filled circles of increasing grey value) for each of the eight 12-week maintenance treatment cycles. Treatment intensity refers to the weighted mean antimetabolite dose intensity (ie, product of weighted mean dose intensities of 6-mercaptopurine and methotrexate; %) mapped against the weighted mean neutrophil count (×10^9^/L) for each 12-week maintenance treatment cycle. The red circle summarizes treatment intensity over 96 weeks of ALL maintenance treatment. Horizontal parallel dash lines indicate target weighted mean neutrophil count range (in this case, 0.75-1.5 × 10^9^/L). Vertical parallel dotted lines indicate target weighted mean antimetabolite dose intensity (in this case, 80%-120%). (C) Descriptive indicators of hematological toxicity requiring treatment interruption. For each hematological toxicity type (in this case, neutropenia and thrombocytopenia), the number and cumulative duration of toxicity episodes, both overall and for the subset of long-duration toxicity episodes (in this case, episodes ≥ 3 weeks duration) is recorded. PLT, platelet count. (D) Descriptive indicators of prescriber compliance with protocol-specified guidelines for STOP (in this case, absolute neutrophil count < 0.5 × 10^9^/L and/or platelet count < 50 × 10^9^/L), REDUCE (in this case, dose reduction by 50% when absolute neutrophil count between 0.5 and 0.75 × 10^9^/L and/or platelet count between 50 and 75 × 10^9^/L), and INCREASE (in this case, 6-mercaptopurine dose increase by 10% when serial neutrophil count ≥ 0.75 × 10^9^/L and platelet count ≥ 75 × 10^9^/L for 8 consecutive weeks) of drug doses, and estimation of the time to the first attempted dose increase (in this case, 11 weeks for the first attempted dose increase of 6-mercaptopurine dose). [*Patient UPN_217 from the Mendeley Acute Lymphoblastic Leukaemia Maintenance therapy dataset, Table S4].

Figure 2 (patient UPN_217) illustrates “titration to tolerance” through serial drug up-titration. Visualization of serial neutrophil counts and prescribed antimetabolite drug doses indicates periodic drug up-titration attempts over 96 weeks of maintenance treatment (Figure 2A), punctuated by treatment interruptions for below-threshold neutrophil counts. Line plots of drug doses indicate that the highest tolerated 6-mercaptopurine and methotrexate dose combination in patient UPN_217 was 500 mg/week and 22.5 mg/week, respectively (see also Table S4). Cycle-specific summary measure (CSSM), that is, visualization of treatment intensity changes over the eight 12-week-long maintenance treatment cycles (Figure 2B) reiterates this finding and suggests that the maximum tolerable antimetabolite dose intensity (ie, the combined dose intensities of 6MP and MTX) in patient UPN_217 is probably between 180% and 240%. The analysis of hematological toxicity rates (Figure 2C), together with the line plots, indicates that periodic drug up-titration was associated with 5 neutropenia episodes (neutrophil count <0.5 × 10^9^/L) that required treatment suspension, including an episode that was of relatively long duration (≥3 weeks). By contrast, drug up-titration was not associated with episodes of thrombocytopenia. The analysis of prescriber compliance with protocol rules for drug titration (Figure 2D) was overall satisfactory, with one unexplained “STOP” decision and one instance of failure to “INCREASE” dose. Dose up-titration was initiated as early as 11 weeks, and as is apparent from Table S3, drug treatment was associated with target-range neutrophil counts for 10 weeks of the 96-week treatment phase (time in target neutrophil range, 10%).

**Figure 3.**
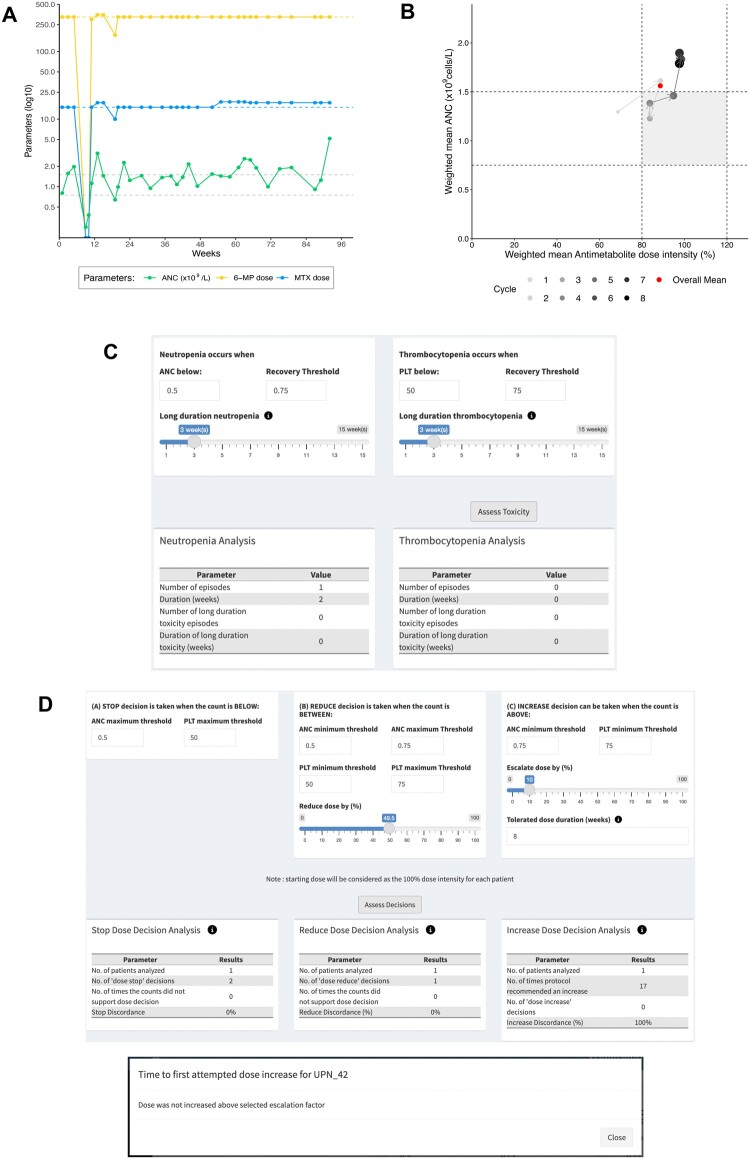
Illustration of absent drug titration in ALL maintenance* (refer text for full details). (A) Longitudinal line plot of blood count (ANC, absolute neutrophil count) and antimetabolite drug doses (6-mercaptopurine [MP] and methotrexate [MTX]) at each dosing visit (weeks 0-96). Horizontal dash lines indicate target neutrophil count range (0.75-1.5 × 10^9^/L) and protocol-recommended doses of 6-mercaptopurine (mg/week) and oral methotrexate (mg/week). (B) Cycle-specific summary measures, that is, weighted means of antimetabolite dose intensity (%; product of weighted mean dose intensities of 6-mercaptopurine and methotrexate) *versus* neutrophil count (*×*10^9^/L), over each 12-week ALL maintenance treatment cycle. (C) Hematological toxicity indicators, including number and duration (in weeks) of episodes of neutropenia (in this case, neutrophil count < 0.5 × 10^9^/L) and thrombocytopenia (in this case, platelet count [PLT] below 50 × 10^9^/L), overall and for the subset of long-duration (in this case, duration ≥ 3 weeks) episodes. (D) Prescriber compliance indicators, including concordance of prescriber decisions with protocol-specified rules for dose STOP (in this case, when neutrophil and platelet count below 0.5 and below 50 × 10^9^/L, respectively), dose REDUCE (in this case, 50% reduction in drug doses when neutrophil count between 0.5 and 0.75 × 10^9^/L and/or platelet count between 50 and 75 × 10^9^/L), dose INCREASE (in this case, 6-mercaptopurine dose increase by 10% when neutrophil and platelet counts consistently ≥ 0.75 × 10^9^/L and ≥ 75 × 10^9^/L, respectively, for 8 consecutive weeks), and Time to First Attempted Dose Increase (in this case, no 6-mercaptopurine dose increase was prescribed). [*Patient UPN_42 from the Mendeley Acute Lymphoblastic Leukaemia Maintenance therapy dataset (Table S5); output from the VIATAMIN application].

**Figure 4.**
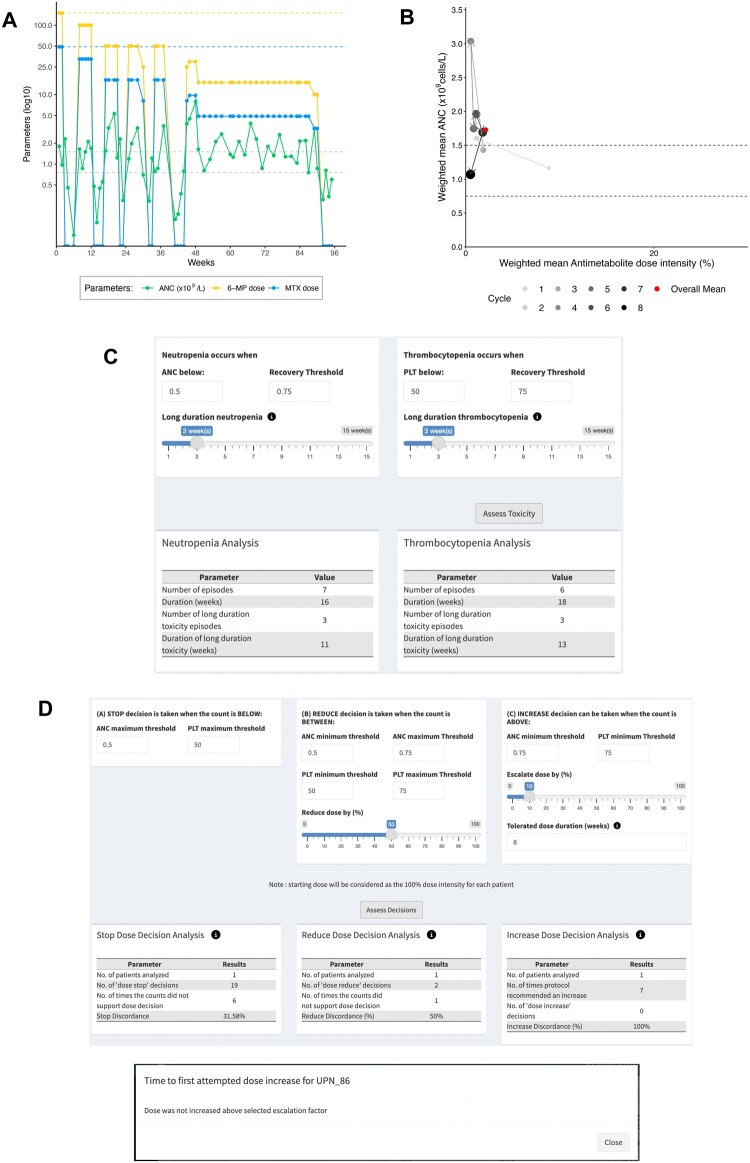
Illustration of drug down-titration in ALL maintenance. The patient had germline heterozygosity for the NUDT15*3 polymorphism (rs116855232, c.415C>T, p. R139C). Full details in the text. (A) Longitudinal line plot of blood count (ANC, absolute neutrophil count) and antimetabolite drug doses (6-mercaptopurine [MP] and methotrexate [MTX]) at each dosing visit (weeks 0-96). Horizontal dash lines indicate target neutrophil count range (in this case, 0.75-1.5 × 10^9^/L) and protocol-recommended doses of 6-mercaptopurine (mg/week) and oral methotrexate (mg/week). (B) Cycle-specific summary measures, that is, weighted means of antimetabolite dose intensity (%; product of weighted mean dose intensities of 6-mercaptopurine and methotrexate) and absolute neutrophil count (*×*10^9^/L), over each 12-week ALL maintenance treatment cycle. (C) Hematological toxicity indicators, including number and duration (in weeks) of episodes of neutropenia (in this case, neutrophil count < 0.5 × 10^9^/L) and thrombocytopenia (in this case, platelet count [PLT] below 50 × 10^9^/L), overall and for the subset of long-duration (in this case, duration ≥ 3 weeks) episodes. (D) Prescriber compliance indicators, including concordance of prescriber decisions with protocol-specified rules for dose STOP (in this case, when neutrophil and platelet count below 0.5 and below 50 × 10^9^/L, respectively), dose REDUCE (in this case, 50% reduction in drug doses when neutrophil count between 0.5 and 0.75 × 10^9^/L and/or platelet count between 50 and 75 × 10^9^/L), dose INCREASE (in this case, 6-mercaptopurine dose increase by 10% when neutrophil and platelet counts consistently ≥ 0.75 × 10^9^/L and ≥ 75 × 10^9^/L, respectively, for 8 consecutive weeks), and Time to First Attempted Dose Increase (in this case, no 6-mercaptopurine dose increase was prescribed). [*Patient UPN_86 from the Mendeley Acute Lymphoblastic Leukaemia Maintenance therapy dataset (Table S6); output from the VIATAMIN application].

Figures 3 and 4 illustrate additional drug titration practices. Figure 3 highlights failure of drug titration (UPN_42, Table S5). Line plots of drug doses (Figure 3A) indicate treatment at unchanged protocol-recommended (100%) doses, with no attempt at drug up-titration. The CSSM plot (Figure 3B) indicates that the weighted mean neutrophil count was above target range (>1.5 × 10^9^/L) for the final 3 cycles of ALL maintenance (36 of 96 weeks, 38%) suggesting opportunity for further antimetabolite dose escalation. This is corroborated by the low reported rates of hematological toxicity (Figure 3C; one 2-week neutropenia episode alone, early in ALL maintenance) and the many missed opportunities (*n* = 17) for dose increase over the 96-week treatment course (Figure 3D).

Figure 4 highlights steady antimetabolite drug down-titration in response to recurring hematological toxicity. Line plot of serial neutrophil counts (Figure 4A) and hematological toxicity rates (Figure 4C) indicate frequent low blood count episodes (neutropenia, 7 episodes; thrombocytopenia, 6 episodes) requiring treatment halt (19 of 96 treatment weeks, Table S6), including 3 long-duration episodes (cytopenia ≥ 3 weeks). Consequently, antimetabolite doses were decreased serially over the first 4 cycles of ALL maintenance treatment, settling finally on treatment with ultra-low doses of 6-mercaptopurine (15 mg/week). The CSSM plot (Figure 4B) indicates a tolerated antimetabolite dose intensity of <10%, with attempted offset through methotrexate dose intensification (Figure 4A, Table S5). The frequent treatment-related hematological toxicity probably influenced prescriber compliance with protocol dosing rules, Figure 4D, accounting for frequent discretionary “STOP” and “REDUCE” dose decisions outside protocol rules (32% and 50% for “STOP” and “REDUCE” dose decisions, respectively). The poor drug tolerance phenotype prompted testing for commonly described constitutional polymorphisms in *NUDT15* and *TPMT*; heterozygosity for the NUDT15*3 polymorphism (rs116855232, c.415C>T, p. R139C) was later diagnosed.

### Cohort-level analysis summarizes drug titration practice in patient groups and enables evaluation of the impact of changes in clinical workflows

**Figure 5.**
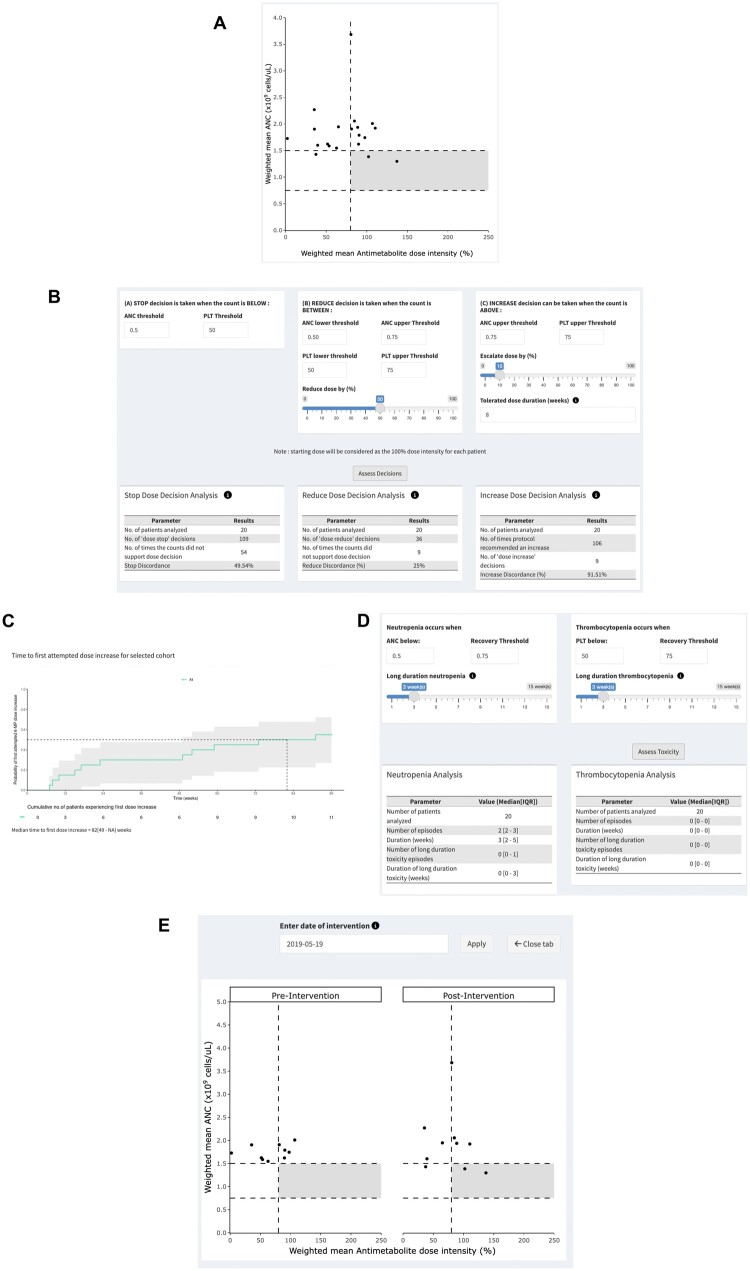
Evaluation of ALL maintenance treatment intensity in patient cohorts: (A) overall and (B) following directed interventions to optimize titration practice. (A) Scatterplot representation of ALL maintenance treatment intensities in a 20-patient cohort. Each filled circle represents the weighted mean antimetabolite dose intensity (x-axis; product of the weighted mean dose intensities of 6-mercaptopurine and methotrexate; %) and weighted mean absolute neutrophil count (y-axis; × 10^9^/L; ANC) in an individual patient. Horizontal dash lines indicate the target weighted mean absolute neutrophil range (ANC, in this case, between 0.75 and 1.5 × 10^9^/L), the vertical dashed line indicates the minimum targeted weighted mean antimetabolite dose intensity (in this case, 80%). The gray-filled region in this example denotes the desired treatment intensity in ALL maintenance. (B) Prescriber compliance indicators, including concordance of prescriber decisions with protocol-specified rules for dose STOP (in this case, when neutrophil and platelet count [PLT] below 0.5 × 10^9^/L and below 50 × 10^9^/L, respectively), dose REDUCE (in this case, 50% reduction in drug doses when neutrophil count between 0.5 and 0.75 × 10^9^/L and/or platelet count between 50 and 75 × 10^9^/L), and dose INCREASE (in this case, 6-mercaptopurine dose increase by 10% when neutrophil and platelet counts consistently ≥ 0.75 × 10^9^/L and ≥ 75 × 10^9^/L, respectively, for 8 consecutive weeks). (C) Cumulative incidence plot indicating median time to first 6-mercaptopurine dose increase (in this case, median of 66 weeks, 95% confidence interval 20 weeks—not evaluable). (D) Hematological toxicity indicators, including number and duration (in weeks) of episodes of neutropenia (in this case, absolute neutrophil count [ANC] < 0.5 × 10^9^/L) and thrombocytopenia (in this case, platelet count [PLT] below 50 × 10^9^/L), overall and for the subset of long-duration (in this case, duration ≥ 3 weeks) episodes. (E) Scatterplot representation comparing ALL maintenance treatment intensity in patient cohorts prior to (“Pre-Intervention,” *n* = 10) and following (“Post-Intervention,” *n* = 10) transition to a hybrid mode (ie, in-person and remote monitoring) of supervision of ALL maintenance treatment (intervention date, 15 May 2019). Horizontal dash lines indicate the target weighted mean absolute neutrophil count range (ANC, in this case, between 0.75 and 1.5 × 10^9^/L), the vertical dash line indicates the minimum targeted weighted mean antimetabolite dose intensity (in this case, 80%). The gray-filled region denotes the desired treatment intensity in ALL maintenance. [*Refer full text and Table S7 for further details; output from the VIATAMIN application].

Figure 5A includes a scatterplot representation summarizing ALL maintenance treatment intensity in each of a 20-patient cohort. For each patient (indicated by a dot in the plot), summary treatment intensity represents a composite of the weighted means of neutrophil count and antimetabolite dose intensity over 96 weeks of ALL maintenance treatment. As shown in the Figure 5A, 10 (50%) patients recorded above-threshold weighted mean antimetabolite dose intensity (≥80% in this example), 2 of whom also recorded weighted mean neutrophil count within target range (in this case, ≤1.5 × 10^9^/L). The analysis of prescriber compliance with protocol drug titration rules (Figure 5B) indicated frequent deviations from blood count-specified thresholds for dose “STOP” (50%), “REDUCE” (25%), and “INCREASE” (92%). As corroboration, the cumulative incidence plot indicates a considerably delayed median time (Figure 5C; 82 weeks; 95% confidence interval, 49-NA) to first drug dose increase. Inadequate drug up-titration is highlighted further by the low rates of hematological toxicity in the cohort (Figure 5D; 2 neutropenia episodes, no thrombocytopenia episodes).

Figure 5E illustrates the utility of the analysis tools in assessing the impact of changes in operational procedures on drug titration practice in ALL·MT. Of the 20-patient cohort above, a sub-cohort of 10 patients (Sub-cohort 2, Table S7A) was managed in a hybrid mode due to COVID-19 restrictions and received two-thirds of their care via email-based advice.^11^ Despite remote supervision, no decline in treatment intensity was observed in this sub-cohort. Scatterplot analysis revealed comparable treatment intensity between Sub-cohorts 1 and 2, with an equal number of patients in both groups achieving target weighted mean antimetabolite dose intensity (≥80%). Supplementary analysis (Table S7B) confirmed these findings, indicating no significant changes in key treatment intensity indicators, including neutrophil count and antimetabolite drug exposure.

## Discussion

The central tenet in ALL maintenance is to maximize systemic exposure to the oral antimetabolite drugs 6-mercaptopurine and methotrexate through the course of treatment. In practice, this is achieved through periodic drug titration to identify and administer the highest tolerated antimetabolite dose combination in individual patients. Implementing this principle in the clinic is a challenge. In the absence of dose titration evaluators, practitioners often prescribe fixed drug doses and opt to only reduce doses or stop treatment in instances of toxicity.

In this report, we discuss the development of simple analytical tools to evaluate the implementation of drug titration precepts in ALL maintenance treatment. This open-access suite of tools, based on the R-programming language, combines data visualization with numerical estimates to evaluate drug titration practice in individual patients and patient cohorts during ALL maintenance treatment. The toolkit includes a user-friendly interactive web application to enable practitioners identify both optimal and variant drug titration practices in individual patients. Analysis begins with data visualization plots, and interpretation of findings is supported by estimates of hematological toxicity rates and the extent of prescriber compliance with drug titration rules (including suspension, reduction and escalation of drug doses).

As highlighted in the Results section, this integrated analysis reveals recurring drug titration patterns that are influenced by distinct patient- and/or prescriber-related factors (Figure S1). In patient cohorts, use of the analysis toolkit facilitates both overall assessment of treatment intensity in ALL maintenance and evaluation of the impact of changes in treatment supervision and clinical workflows on maintenance treatment intensity.

The analysis toolkit has been developed using readily accessible demographic and clinical data, including patient body surface area, serial blood count values, dose advice dates, and dose prescriptions. The toolkit is protocol-agnostic and accommodates variations in protocol-specific blood count thresholds for drug titration practice across different ALL maintenance regimens. The current version does not include additional variables that influence drug titration practice. These variables are either less readily available or less consistently documented and include non-hematological toxicity events (such as thiopurine-associated liver toxicity), assessments of patient adherence, and information on molecular biomarkers (such as pharmacogenomic determinants and pharmacological markers). These variables will be incorporated in future iterations.

The use of this toolkit in our practice has yielded instructive insights into understanding and explaining the variations in drug titration practice and highlighting potential strategies to address suboptimal drug titration. Toolkit observations have served as a means of auditing drug titration practice and as a training tool for practitioners. By enabling the visualization of drug titration practices, the toolkit allows practitioners to identify major deviations from protocol-specified drug titration rules. This is especially useful for less experienced clinicians who often hesitate to escalate antimetabolite dosages to the limits of tolerance. Incorporation of toolkit elements in forthcoming automated dose advice systems^12^^,^^13^ will thus facilitate real-time monitoring of drug titration practice in ALL maintenance, enabling standardization and audit of ALL maintenance treatment across cancer centers.

## Supplementary Material

ooae089_Supplementary_Data

## Data Availability

The allMT R package is freely available on CRAN at https://cran.r-project.org/web/packages/allMT/index.html. The dataset is available at https://data.mendeley.com/datasets/775hs9wrb5/1.
