# Supplementary material for: Analysis toolkit for evaluation of drug titration practice in acute lymphoblastic leukemia maintenance
Source: JAMIA Open. 2024 Sep 13;7(3):ooae089. doi: 10.1093/jamiaopen/ooae089 (PMC11398893; doi:10.1093/jamiaopen/ooae089)
Supplement: ooae089_Supplementary_Data [file ooae089_supplementary_data.zip › Supplementary.pdf]

SUPPLEMENTARY INFORMATION

Abbreviations

6MP	6-Mercaptopurine
ALL	Acute lymphoblastic leukemia
ANC	Absolute Neutrophil Count
Hb	Hemoglobin
MT	Maintenance therapy in ALL
MTX	Methotrexate
PLT	Platelet count

Drug titration in ALL maintenance

At the start of the maintenance treatment phase, the antimetabolite drugs 6MP and MTX are administered at protocol-specified doses. Over the course of maintenance treatment, antimetabolite doses are titrated upwards periodically (typically, by 10-25% every 6-8 weeks) to maintain blood counts at safely low target ranges (ANC between 0·75 and 1·5 × 10^9/L; PLT ≥ 75 × 10^9/L in the ICiCLe-ALL-14 treatment protocol). Doses of antimetabolite drugs are increased sequentially (6MP dose increase followed 2-4 weeks later by MTX dose increase) to maintain parity in dose intensities of the two drugs (dose intensity describes drug dose as a proportion of the protocol-specified start dose). Drug treatment is suspended, or doses reduced when blood counts drop and/or when clinical toxicity is observed. Treatment is resumed following recovery from toxicity, typically at previously tolerated dose levels, and upward dose titration is often reattempted after a while. A subset of patients do not tolerate protocol-specified antimetabolite doses and require serial dose decrements to maintain safely low blood counts and/or to avoid clinical toxicity (Supplementary Table S1).

Data recording and MT terminology

During the 2 years of MT, periodic blood count tests are performed, and patients receive advice on 6MP and MTX doses. Although varying by treatment protocol, dosing decisions of 6MP and MTX are typically influenced by the blood neutrophil and platelet counts (ANC, absolute neutrophil count; PLT, platelet count) and additionally, the hemoglobin (Hb) level (and sometimes, the white blood count).

Supplementary Table S2 highlights a typical data structure for recording serial blood counts and drug doses during MT in a patient treated on the ICiCLe-ALL-14 protocol. In this protocol, the ~2 years (96 weeks) of MT is divided into eight 12-week blocks, each block referred to as an MT Cycle (i.e. MT Cycles 1-8)

Each row of Table S1 should be interpreted as follows: patient with unique identification number UPN_138 visited the hospital during week 1 of cycle 1 of MT treatment, had a blood count test done on 17 November 2016, and the results indicated ANC and PLT values of 1·56 × 10^9/L and 404 × 10^9/L respectively. Based on the blood count values, the patient was prescribed 6MP at 700 mg/week and MTX at 35 mg/week. The columns 'MP_adj' and 'MTX_adj' represent the dose intensities of 6MP and MTX respectively (described in the section below). Supplementary Table S2 is the default data structure that the *allMT* package uses for analysis.

Based on Supplementary Table S2, we define terminologies used in subsequent sections

1. *Prescribed weekly doses of 6MP and MTX*
   This refers to the weekly prescribed dose of 6MP and MTX. In the case of patient UPN_138, the prescribed weekly doses of 6MP and MTX on week 14 (Cycle 2, week 2; 16 February 2017) were 700 mg and 35 mg respectively. In week 33 (Cycle 3, week 9; 29 June 2017), the prescribed weekly doses of 6MP and MTX were 300 mg and 10 mg respectively.
2. *First visit dose*
   The first visit dose represents the starting doses of 6MP and MTX. For UPN_138, the start (or first visit) doses of 6MP and MTX were 700 mg/week and 35 mg/week respectively. These starting doses were 91% and 96% of the protocol-recommended doses for 6MP (770 mg/week) and MTX (36 mg/week) respectively.
3. *Dose intensities of 6MP and MTX*
   Dose intensity reports prescribed 6MP and MTX doses as a proportion of the protocol-recommended (100%) dose of each drug. For UPN_138, in week 33 (Cycle 3, week 9; 29 June 2017), the 6MP dose intensity was 39% (300 mg ÷ 770 mg) and the MTX dose intensity was 27% (10 mg ÷ 36 mg).
4. *Antimetabolite dose intensity (AnMtb)*
   The antimetabolite dose intensity refers to the product of the respective dose intensities of 6MP and MTX. The product function reflects the synergistic therapeutic activity of the two antimetabolite agents during MT.
5. *Weighted means*
   Weighted means of ANC and antimetabolite dose intensity (AnMtb) are determined using weights that represent the intervals (in weeks) between two consecutive dose advice reviews, i.e., for each visit (review) 'i':
   the set {v_i, wk_i, anc_i, plt_i, $6mp_i$, mtx_i} represents information on the treatment (v_i,), MT treatment week (wk_i), neutrophil and platelet counts (anc_i, plc_i), and doses of 6MP and MTX in mg/week ($6mp_i$, mtx_i).

Weight for weighted means (w_i) represents the interval in weeks between two consecutive dose reviews and represents the mathematical weight for determining weighted averages. The review interval w_i ranges from 1-8 weeks; observations from review intervals exceeding 8 weeks are not included in the analysis since these long intervals between reviews do not accurately capture or reflect the therapeutic effect of 6MP and MTX doses on blood counts. For the ith review, the weight, w_i, was computed as $w_i = wk_{i+1} - wk_i$. For the ith review, the dose intensity d_i for either 6MP or MTX is represented as [d_i = (prescribed dose at i^{th} review) / (first visit dose)]

In view of the synergistic therapeutic effects of 6MP and MTX in maintenance, the product of the dose intensities of 6MP and MTX were considered to better represent treatment with these agents during maintenance. This product, referred to as the antimetabolite dose intensity (d_AnMtb), is represented as [$d\_AnMtb_i = d\_6MP_i * d\_MTX_i$] where d_6MP_i and d_MTX_i indicate the dose intensities of 6MP and MTX respectively at the ith review. Table A describes the variables and their definition.

Table A: Definition and notation of derived variables

Derived variable	Definition	Notation
Weight (w_i)	Interval between reviews	$\{w_1, w_2, \dots wn_{-1}\}$
Dose intensity, 6MP (d_6MP_i)	Ratio of prescribed to protocol-recommended dose of 6MP	$\{d\_6mp_1, d\_6mp_2, \dots d\_6mp_{n-1}\}$
Dose intensity, MTX (d_MTX_i)	Ratio of prescribed to protocol-recommended dose of MTX	$\{d\_mtx_1, d\_mtx_2, \dots ,d\_mtx_{n-1}\}$
Antimetabolite dose intensity (d_AnMtb_i)	Product of dose intensities of 6MP and MTX	$\{d\_AnMtb_1, d\_AnMtb_2, \dots, d\_ANMtb_{n-1}\}$

Using the above derived variables and the set of observed variables $\{v_i, wk_i, anc_i, plc_i, 6mp_i, mtx_i\}$ at each review, we calculate weighted means of serial neutrophil counts (*wm*ANC) and antimetabolite dose intensities (*wm*AnMtb). Weighted mean refers to the average of weighted observations. Information from these weighted means are then integrated to summarise maintenance dosing practice for each patient through maintenance. Table B describes the formula for the same.

Table B: Definition for weighted means calculation for ANC and AnMtb

**Variable**	**Definition**
Weighted mean ANC *(wmANC)*	$\frac{w_1 anc_1 + w_2 anc_2 + \cdots + w_{n-1} anc_{n-1}}{w_1 + w_2 + \cdots + w_{n-1}}$
Weighted Mean AnMtb *(wmAnMtb)*	$\frac{w_1 d\_AnMtb_1 + w_2 d\_AnMtb_2 + \cdots + w_{n-1} d\_AnMtb_{n-1}}{w_1 + w_2 + \cdots + w_{n-1}}$

R Functions in the allMT R package

1. **Data recording for MT**

   In a hospital-based practice, data required for audit of ALL MT would likely need to be collated from diverse sources. The *allMT* package provides a sample copy of an Excel workbook that a hospital could repurpose for data recording. Supplementary Table S3 is a sample workbook used at the Tata Medical Centre (TMC) Kolkata to record MT information. The workbook can be downloaded after installing the *allMT* package with the following commands:

```
pat_data <- system.file("extdata/tmc_data/", "UPN_916.xls", package =
"allMT")
dest_path <- getwd()
file.copy(pat_data, dest_path)
```

2. **Maintenance therapy raw data transformation**

   allMT functions require that the data for any given patient follow the format described in Supplementary Table S2. Two functions are provided to reorganize any hospital-specific MT data into the required Table S1 data structure. The main reason for providing the transformation function is (i) to have uniform data at single or multiple sites and (ii) to re-use the data thus recorded for any secondary or future analysis, or as a part of a research study.

   Two methods are provided to convert MT data into a standard format (Table 1) that is used by the rest of the functions of the package. These are

a. convert_tmc_format()

   This converts the TMC workbook (described earlier) into the required format (Supplementary Table S2) for a given patient. Users need to provide the path to the folder containing patients' MT data workbooks along with a destination folder path to save the new standard format files. Additionally, users need to provide the body surface area-based protocol doses of 6MP and MTX as per protocol, to compute dose intensities.

   Usage:

```
convert_tmc_format(inputpath_to_excelfolder,exportpath_to_csvfolder,
daily_mp_dose, weekly_mtx_dose)
```

b. convert_external_format()

   This function converts alternate data recording formats used at different treatment centres. Users will need to ensure that for each patient, data is present in an Excel/csv file with column names 'Cycle', 'Dates', 'Weeks', 'ANC', 'PLT', 'Hb', 'MP', 'MP_adj', 'MTX' and 'MTX_adj' in any order. Here, MP_adj and MTX_adj columns represents the dose intensities of 6MP and MTX dose, as per the respective institutional treatment

protocol (outlined in the section on MT Terminology). The paths to the folder containing these files, and the folder to which converted files should be saved must be provided to the function. It will convert each sheet into the Supplementary Table S1 format and save it in the destination folder.

Usage:

```
convert_external_format(inputpath_to_excelfolder,
exportpath_to_csvfolder, pat_data_file_path, daily_mp_dose,
weekly_mtx_dose)
```

3. **Analyzing maintenance therapy data: Visualization**

A. Individual patient level

As the patient progresses through 2 years of MT, the volume of longitudinal data, including serial blood counts and dose prescription values, becomes difficult to understand and analyze at one go. The two functions below can be used to track the patient's treatment and treatment response in real time in any hospital setting.

1. plot_progression()

   The function creates a line graph of serial neutrophil counts (ANC) and the accompanying prescribed doses (mg/week) of 6MP and MTX. The function requires specification of the path to the patient's csv file (created with use of the data transformation functions), the protocol-specified target range for ANC (e.g. between $0{\cdot}75 \times 10^9$/L and $1{\cdot}5 \times 10^9$/L in the ICiCLe-ALL-14 treatment protocol) and the laboratory unit for ANC values (e.g., 10^3/μL, 10^3/mm^3, 10^9/L).

   Usage

   ```
   plot_progression(input_file_path, anc_range, unit)
   ```

2. summarize_cycle_progression()

   The function plots the weighted mean of antimetabolite dose intensity against ANC for each of the eight MT cycles to understand the dose titration practice across MT. Arguments are the same as the `plot_progression()` function.

   Usage:

   ```
   summarize_cycle_progression(input_file_path, anc_range, unit)
   ```

B. Cohort level

Evaluation of the overall antimetabolite dose titration practice during MT can be evaluated in a cohort of patients using the following functions:

1. summarize_cohortMT()

   This function summarises weighted mean anti-metabolite dose intensity (wmAnMtb) and ANC (wmANC) over the course of MT for each patient in a cohort and generates a scatter plot. Input arguments include the path to the folder with csv

files (generated using the data transformation function), the arguments used for the plot_progression() function and the reference dose intensity threshold selected
Usage:
```
summarize_cohortMT(input_files_path, anc_range, unit,
dose_intensity_threshold)
```

2. compare_cohorts()
   The function is similar to the above with the additional feature of comparing multiple cohorts. It can be used to compare dose titration practice between two or more MT cohorts to evaluate impact of MT-related interventions or variations between treatment centers.
   The first four arguments of the function are the same as `summarize_cohortMT()`. The cohorts may be compared in one of 2 ways ("M1" or "M2"), determined by the "method" argument. "M1" compares cohorts that started MT before or after a particular date and "M2" compares MT between pre-determined groups. Method "M1" requires a date (yyyy-mm-dd) for the '`intervention_date`' argument. Method M2 requires the path to the Excel file containing "group" information. The Excel file should have two columns - "ID" and "Group".
   Usage:
```
compare_cohorts(input_files_path, unit, anc_range,
dose_intensity_threshold, method, intervention_date,
group_data_path)
```

4. Analysis of compliance with protocol recommendations for antimetabolite dose modifications

Dose titration during MT drug dosing is based on protocol-defined rules. The functions in this section evaluate dose decisions of practitioners against protocol recommendations for dose suspension, dose reduction and dose increase. These functions may be used at any given point of time during the 2 years of MT.

1. assess_stop_doses()
   The function returns the number of times STOP decisions (suspension of 6MP and MTX treatment) were supported by blood count parameters. Depending upon whether the dose decisions are assessed for an individual patient or a cohort, the path to csv file/files (following data transformation) must be provided. In addition, ANC, PLT and Hb thresholds below which doses are stopped should be specified. A minimum one parameter (ANC, PLT and Hb) is required for analysis, else an error message will be displayed.
   Usage:

```
assess_stop_doses(input_files_path,  anc_threshold = NA,
plt_threshold = NA, hb_threshold = NA)
```

2. assess_reduced_doses()
The function evaluates the number of times REDUCE dose decisions were supported by blood count parameters. The 'input_files_path' argument is the same as the assess_stop_doses() function. Dose reduction thresholds for ANC ('anc_threshold'), PLT ('plt_threshold') and Hb ('hb_threshold') should be provided as (a,b) where a and b represent the upper and lower limits between which doses are reduced. The last argument of this function is the 'dose reduction factor' which indicates the magnitude of dose reduction (as a proportion or percentage of the dose prescribed in the preceding visit).
Usage:
```
assess_reduced_doses(input_files_path, anc_range = NA, plt_range =
NA, hb_range = NA, reduction_factor)
```

3. assess_increased_doses()
The function assesses the number of times serial blood counts provided opportunity for drug dose INCREASE. The first argument is the same as the above two functions. The subsequent parameters indicate the specified minimum duration ('tolerated_dose_duration') when above-threshold blood counts (i.e., ANC, PLT, Hb values) were recorded. The 'escalation_factor' refers to the magnitude of dose increase, as a percentage of the preceding dose
Usage:
```
assess_increased_doses(input_files_path, anc_threshold = NA,
plt_threshold = NA, hb_threshold = NA, escalation_factor,
tolerated_dose_duration)
```

4. Time to first 6MP dose increase
This function reports the time to the first prescribed increase in the dose of 6MP (increase by a minimum of 10% from the 100% start dose of 6MP) during MT. The function described in this section may be used for a single patient or a cohort.
Usage:
```
time_to_first_dose_increase(input_files_path, escalation_factor)
```

5. Analysis of hematological toxicities

This section provides functions to assess hematological toxicities during MT and may be used during or after MT treatment in an individual patient or for a cohort.

The assess_anemia(), assess_neutropenia(), and assess_thrombocytopenia() functions report the total number and cumulative duration of episodes of anemia, neutropenia, and thrombocytopenia respectively for an individual patient or cohort. Additionally, the functions report the number and total duration of episodes of prolonged hematological toxicity. When analyzing a cohort, the above results are reported as median and interquartile range.

All three functions have the first argument as the path to a csv file (for a single patient) or multiple csv files (for a cohort), following the data transformation function. The second argument has a common format of (a,b) where 'a' is the threshold representing anemia, neutropenia and thrombocytopenia states, and 'b' is the threshold blood count value above which the toxicity conditions are considered to be recovered. The last argument is the specified duration of prolonged hematological toxicity

Usage:

```
assess_anemia(input_files_path, hb_range, duration_hb = NA)
assess_neutropenia(input_files_path, anc_range, duration_anc = NA)
assess_thrombocytopenia(input_files_path, plt_range, duration_plt = NA)
```

The source code for the *allMT* library is available at https://github.com/tmungle/allMT. The link https://tmungle.github.io/allMT/reference/index.html provides instructions on the use of the various functions in the allMT package

VIsualisation & Analysis Tool in ALL MaINtenance (VIATAMIN) Shiny Application

To enable clinical practitioners, use the above functions for visualization and analysis of the maintenance phase of ALL (Supplementary Figure S1), we created VIATAMIN[2], an interactive web-based application developed using the R/Shiny package. The user-friendly application allows use of all functions in the *allMT* R package, including graphical and descriptive analyses for individual patients or a cohort of patients.

1. ***VIATAMIN* features**

   When launched, the *VIATAMIN* application opens with a 'Get started' page, which explains how to use the application, including points to keep in mind when using the application, exemplar input data, developer contact information and references. At the right of the page is a 'Help Section' with 'Further information' that includes a reference manual and a video explainer of application use.

2. ***Help* tab**

   The *Get Started* page and all analysis tabs have a *Help* button that leads to the Help section. This section contains a user manual detailing input parameters and describing output plots or analysis tables with exemplars.

A sidebar to the left of the 'Get started' page contains selection options for the type of analysis, i.e., at an individual patient level, or at cohort level. The required inputs for the selected analysis option will be shown along with a 'data upload' section and 'submit' button.

For an individual patient, analysis functions include (i) *Longitudinal progression*, (ii) *Serial treatment intensity*, (iii) *Assess dose decisions* and (iv) *Assess haematological toxicity* functions.

For a patient cohort, analysis functions include (i) *Cohort summary measures*, (ii) *Compare cohorts*, (iii) *Assess dose decisions* and (iv) *Assess haematological toxicity*.

Once data is uploaded and one or more analysis functions are selected, new tabs will open reporting results of the selected analyses. Unlike the *allMT* R package, the application is unable to convert patient data records into the required format (allMT functions: `convert_tmc_format` or `convert_external_format`). Users will need to upload patient data as a processed excel (.xls, .xlsx or .xlsm), or csv file (Supplementary Table S2).

3. ***Patient data* tab**

   Exclusive to the MT analysis in an individual patient, the 'patient data' tab will open when an individual patient's data file is uploaded, and an analysis function is selected. It displays the uploaded patient file, which serves as reference information when interpreting visual or quantitative outputs in the application.

4. ***Plots* tab**

   Longitudinal plots of serial blood counts and the corresponding 6MP and MTX drug doses and antimetabolite dose intensities are used to analyze dose titration practice during MT, both for an individual patient and for a patient cohort. For an individual patient, this includes visualization of ANC values and the prescribed weekly doses of 6MP and MTX at each visit (VIATAMIN function: *Longitudinal progression*) together with the longitudinal plot of the weighted means of ANC and antimetabolite dose intensities for each of the eight MT cycles (VIATAMIN function: *Serial Treatment Intensity*). At the cohort level, a scatter plot indicates MT treatment intensity in individual patients, as a composite of the weighted means of ANC and antimetabolite dose intensity (VIATAMIN function: *Summary Measures*). The scatter plot representation could also be used to compare MT treatment intensity between cohorts (VIATAMIN function: *Compare cohorts*).

5. ***Dose Decisions* tab**

   This tab allows assessment of three types of dosing decisions – 'stop', 'reduce' and 'increase' drug dose (VIATAMIN function: *Dosing Decisions*). Users would need to

enter the recommended blood count parameters for each of these dosing decisions and the results will indicate the discordance between protocol-recommended decisions and the actual decisions taken by physicians over the course of MT treatment, both in individual patients and patient cohorts. Discordance is determined by estimating the number of stop/reduce/increase dose decisions in practice against the number specified by the recommended blood count parameters, reported as a percentage. The '*Assess time to dose increase*' VIATAMIN function in this tab also analyses the time taken for the first increase in 6MP dose following start of MT. This is a measure of the timeliness of dose up-titration during MT.

6. ***Toxicity* tab**
   In this tab, users can specify protocol-defined blood count thresholds for one or more hematological toxicities including neutropenia, thrombocytopenia and anemia. For each type of hematological toxicity, the tab reports the number of toxicity episodes, the cumulative duration of toxicity, the number of prolonged or 'long duration' toxicity episodes, and the cumulative duration of these 'long duration' toxicity episodes. In the case of patient cohorts, these numbers are reported as median and interquartile range.

7. **Reference manual and tutorial**
   The *VIATAMIN* application includes a reference manual, accompanied by a video tutorial on the use of the *VIATAMIN* application ('How to: ALL MT Analysis with VIATAMIN' at https://shorturl.at/aKPRX; last accessed, 26 March 2024)

References

1. Mungle T, Mahadevan M, Gogoi MP, et al. "Acute lymphoblastic leukaemia maintenance therapy dataset", Mendeley Data, V1, 2023 doi: 10.17632/775hs9wrb5.
2. **VI**sualisation & **A**nalysis **T**ool in **A**LL **M**a**IN**tenance (VIATAMIN). https://ananyam.shinyapps.io/VIATAMIN/ (last accessed, 26 March 2024)

Supplementary Table S1: Dose titration decisions in ALL Maintenance (based on the ICiCLe-ALL-14 Indian Collaborative Childhood Leukaemia 2014 multicentre treatment protocol for newly diagnosed acute lymphoblastic leukemia)

Treatment decision	Clinical & Laboratory parameters	Target blood count values[a]			Antimetabolite dose prescription
		Hb (g/dL)	ANC (× 10^9/L)	PLT (× 10^9/L)	
'START'	Completion of ALL intensive phase<br>Recovery of blood counts<br>Clinically well	≥ 8	≥ 1	≥ 100	Start 6MP and MTX at 100% doses[b]
'CONTINUE'	Blood counts within target range<br>Clinically well, adherent to treatment<br>6-8 weeks since last dose change	≥ 7	≥ 0·75	≥ 75	Continue at previously prescribed doses
'INCREASE'	Blood counts within target range<br>No apparent declining trend in blood counts<br>Clinically well, adherent to treatment<br>Completed 6-8 weeks at a given dose level	≥ 8	≥ 0·75	≥ 75	Increase 6MP dose by 10-25%<br>Matching increase in MTX 2-4 weeks later
'STOP'	Blood counts below target range<br>And/or clinical toxicity[c]	< 7	< 0·5	< 50	Stop 6MP and MTX until recovery
'REDUCE'	Blood counts below target range	·	≥ 0·5; < 0·75	≥ 50; < 75	Reduce 6MP and MTX doses<br>Reduce by 50% of previously prescribed doses
'RESUME'	Recovery of blood counts<br>And/or resolution of clinical toxicity	≥ 7	≥ 0·75	≥ 75	Resume 6MP and MTX treatment[d]

[a]Blood count thresholds used in the ICiCLe-ALL-14 clinical protocol for newly diagnosed paediatric ALL; blood counts tested ideally every 2 weeks

[b]100% doses based on protocol-recommended start doses for oral 6MP and MTX; ICiCLe-ALL-14 protocol, 6MP 60mg/m^2/day (420 mg/m^2/week); MTX, 20 mg/m^2/week

[c]Clinical toxicity, including infections and recurring non-infection drug toxicities (mucocutaneous, hepatic, pancreatic, gastrointestinal toxicities)

[d]Antimetabolites may be resumed at previously prescribed doses or at a previously tolerated dose combination

6MP, 6-mercaptopurine; ALL, acute lymphoblastic leukaemia; Hb, haemoglobin; ANC, absolute neutrophil count; MTX, methotrexate; PLT, platelet count

ICiCLe-ALL-14: Indian Collaborative Childhood Leukaemia treatment protocol (2014) for newly-diagnosed acute lymphoblastic leukaemia

ALL·MT includes *P. jirovecii* prophylaxis (trimethoprim-sulphamethoxazole), periodic intrathecal treatments (with MTX) and in some protocols, steroid-vincristine pulses

Supplementary Table S2: ALL Maintenance data table of patient UPN_138 (available at the Acute Lymphoblastic Leukaemia maintenance therapy dataset from Mendeley Data).

[ANC, absolute neutrophil count, × 10^9/L; PLT, platelet count; × 10^9/L Hb, hemoglobin in g/dL; MP, 6-mercaptopurine weekly dose, mg/week; MP_adj, 6-mercaptopurine dose intensity (prescribed / protocol dose), %; MTX, oral methotrexate weekly dose, mg/week; MTX_adj, oral methotrexate dose intensity (prescribed / protocol dose), %; bold highlight indicate visits where drug treatment was withheld]

**Treatment** **Cycle**	**Date** dd/mm/yyyy	**Treatment** **Week**	**ANC** × 109/L	**PLT** × 109/L	**Hb** g/dL	**MP** mg/week	**MP_adj** %	**MTX** mg/week	**MTX_adj** %
1	17/11/2016	1	1.56	404	11.9	700	91	35	96
1	01/12/2016	3	1.80	272	11.9	700	91	35	96
1	15/12/2016	5	1.78	210	12.6	700	91	35	96
1	29/12/2016	7	0.81	149	12.8	700	91	35	96
**1**	**05/01/2017**	**8**	**0.47**	**84**	**12.2**	**0**	**0**	**0**	**0**
**1**	**12/01/2017**	**9**	**0.58**	**146**	**12.7**	**0**	**0**	**0**	**0**
**1**	**19/01/2017**	**10**	**0.39**	**184**	**13.1**	**0**	**0**	**0**	**0**
1	02/02/2017	12	3.30	191	13.9	700	91	35	96
2	16/02/2017	14	1.40	136	13.1	700	91	35	96
**2**	**02/03/2017**	**16**	**0.21**	**18**	**10.7**	**0**	**0**	**0**	**0**
**2**	**09/03/2017**	**17**	**0.23**	**70**	**10.0**	**0**	**0**	**0**	**0**
**2**	**16/03/2017**	**18**	**0.50**	**187**	**9.6**	**0**	**0**	**0**	**0**
2	23/03/2017	19	1.53	274	11.2	350	46	17.5	48
2	06/04/2017	21	5.05	264	11.4	500	65	17.5	48
2	20/04/2017	23	2.06	228	12.4	500	65	17.5	48
3	04/05/2017	25	2.67	157	11.7	600	78	17.5	48
3	18/05/2017	27	1.44	118	11.0	600	78	17.5	48
3	01/06/2017	29	0.66	105	10.6	300	39	10	27
**3**	**08/06/2017**	**30**	**0.56**	**92**	**9.8**	**0**	**0**	**0**	**0**
3	15/06/2017	31	1.02	193	9.9	300	39	10	27
3	29/06/2017	33	2.78	213	10.9	300	39	10	27
3	13/07/2017	35	7.02	217	10.8	300	39	10	27
4	27/07/2017	37	4.97	237	11.8	450	59	15	41
4	10/08/2017	39	5.03	189	11.7	450	59	15	41
4	24/08/2017	41	3.86	165	12.6	450	59	25	68
4	07/09/2017	43	1.45	66	10.4	225	29	12.5	34
4	14/09/2017	44	2.67	106	10.4	225	29	12.5	34
4	28/09/2017	46	1.74	166	9.8	225	29	12.5	34
4	05/10/2017	47	2.52	101	9.4	225	29	12.5	34
4	12/10/2017	48	2.96	105	8.3	225	29	12.5	34
5	26/10/2017	50	4.53	105	8.6	225	29	15	41
**5**	**02/11/2017**	**51**	**1.98**	**85**	**7.2**	**0**	**0**	**0**	**0**
**5**	**09/11/2017**	**52**	**3.11**	**112**	**7.9**	**0**	**0**	**0**	**0**
5	23/11/2017	54	3.25	160	9.2	225	29	12.5	34
5	30/11/2017	55	3.23	254	8.6	225	29	12.5	34
5	14/12/2017	57	3.50	153	8.2	250	33	12.5	34
5	21/12/2017	58	3.51	117	8.6	250	33	15	41
5	04/01/2018	60	2.28	127	8.1	250	33	15	41
6	18/01/2018	62	1.57	75	6.9	125	16	7.5	20
6	01/02/2018	64	1.74	196	11.5	125	16	7.5	20
6	15/02/2018	66	5.11	285	11.8	125	16	7.5	20
6	22/02/2018	67	4.76	229	12.3	200	26	10	27
6	08/03/2018	69	4.09	227	12.2	200	26	20	55
6	22/03/2018	71	3.08	104	11.3	200	26	20	55
7	05/04/2018	73	2.75	106	10.0	200	26	25	68
7	19/04/2018	75	3.43	131	9.8	200	26	25	68
7	03/05/2018	77	4.06	270	11.1	200	26	25	68
7	17/05/2018	79	3.06	190	12.9	225	29	25	68
7	31/05/2018	81	2.36	217	12.9	225	29	25	68
7	14/06/2018	83	3.33	102	12.0	225	29	30	82
8	28/06/2018	85	3.02	158	12.4	275	36	30	82
8	12/07/2018	87	2.94	161	12.2	275	36	35	96
8	26/07/2018	89	1.78	78	11.2	275	36	35	96
**8**	**09/08/2018**	**91**	**1.40**	**28**	**9.6**	**0**	**0**	**0**	**0**
8	16/08/2018	92	2.80	260	10.4	275	36	35	96
8	23/08/2018	93	1.30	190	8.9	375	49	35	96
**8**	**30/08/2018**	**94**	**1.30**	**49**	**7.9**	**0**	**0**	**0**	**0**
8	06/09/2018	95	2.30	97	8.0	225	29	25	68

Supplementary Table S3:

Sample data table for longitudinal recording of treatment details for each of the eight 12-week maintenance treatment cycles

**Treatment Cycle**	**Hospital Number**				**Name**				**Date of Birth**			
Height (cm)												
Weight (Kg)												
BSA (m^2)	6-Mercaptopurine		60	mg/m^2	Methotrexate		20	mg/m^2	IT MTX		As per age	
Date												
Treatment week	**1**	**2**	**3**	**4**	**5**	**6**	**7**	**8**	**9**	**10**	**11**	**12**
Neutrophil Count (10^9/L)												
Platelet Count (10^9/L)												
Haemoglobin (g/dl)												
6-Mercaptopurine												
6-Mercaptopurine%												
Oral Methotrexate												
Methotrexate%												
IT Methotrexate												
TMP-SMX												
Comments												

GENERAL INSTRUCTIONS

6-Mercaptopurine (6MP) 60 mg/m^2, oral, to be taken in the evening on empty stomach (no food 1 hour before and after taking 6MP); avoid taking with milk or milk products

Methotrexate (MTX) 20 mg/m^2/week, oral, to be taken once a week on a fixed day together with 6MP; omit in the week of intrathecal (IT) methotrexate treatment

Trimethoprim-Sulphamethoxazole (TMP-SMX) twice daily on two consecutive days every week till 6 weeks after end of treatment. Dose: BSA $<0.75m^2$= 240mg; ≥ 0.75 - $<1m^2$ =

Objective is to maintain absolute neutrophil count (ANC) between 0.75 and 1.5 × 10^9 /L and platelet count (PLT) above 75 × 10^9 /L

If ANC between 0.5 and 0.75 × 10^9/L and/or PLT between 50 and 75 × 10^9 /L, decrease dose of 6MP and oral MTX to 50% of the prescribed dose.

If ANC < 0.5 × 10^9/L and/or PLT ≤ 50 × 10^9/L, stop 6MP and MTX. Consider weekly blood count check and restart once ANC ≥ 0.75 × 10^9/L and Platelets ≥ 75 × 10^9/L

If blood counts persistently low for 3 weeks without therapy, stop TMP-SMX. Monitor for recovery. Consider bone marrow tests to assess disease remission status

Supplementary Table S4: ALL maintenance data table of patient UPN_217 illustrating up-titration of antimetabolite doses to tolerance. The highest tolerated antimetabolite dose combination (grey highlight) was 500 mg/week and 22·5 mg/week of 6-mercaptopurine and oral methotrexate respectively (grey highlight). Entries in bold highlight dose suspension decisions. (available at the Acute Lymphoblastic Leukaemia maintenance therapy dataset from Mendeley Data)

Treatment Cycle	Date (dd/mm/yy)	Treatment week	ANC ($\times 10^9$/L)	PLT ($\times 10^9$/L)	6MP (mg/week)	6MP_adj (%)	MTX (mg/week)	MTX_adj (%)
1	11/01/2018	1	2.2	272	350	103	15	93
1	25/01/2018	3	3.5	335	350	103	15	93
1	08/02/2018	5	3.3	263	350	103	15	93
1	22/02/2018	7	2.6	288	350	103	15	93
1	08/03/2018	9	2.7	283	350	103	15	93
1	22/03/2018	11	2.4	337	400	118	15	93
2	05/04/2018	13	2.0	305	400	118	15	93
2	26/04/2018	16	1.9	358	400	118	15	93
2	03/05/2018	17	1.7	272	400	118	17.5	108
2	17/05/2018	19	2.5	337	400	118	17.5	108
2	31/05/2018	21	2.8	297	400	118	17.5	108
2	14/06/2018	23	4.0	281	400	118	17.5	108
3	28/06/2018	25	3.2	279	500	147	17.5	108
3	12/07/2018	27	2.4	269	500	147	22.5	139
3	26/07/2018	29	2.4	274	500	147	22.5	139
3	09/08/2018	31	2.3	288	500	147	22.5	139
3	30/08/2018	34	3.1	286	625	184	22.5	139
3	13/09/2018	36	2.3	267	625	184	27.5	170
4	27/09/2018	38	1.4	296	625	184	27.5	170
4	11/10/2018	40	0.9	214	625	184	27.5	170
**4**	**01/11/2018**	**43**	**0.5**	**224**	**0**	**0**	**0**	**0**
**4**	**08/11/2018**	**44**	**0.6**	**218**	**0**	**0**	**0**	**0**
**4**	**15/11/2018**	**45**	**0.3**	**336**	**0**	**0**	**0**	**0**
4	22/11/2018	46	3.8	241	625	184	27.5	170
4	06/12/2018	48	3.7	274	625	184	30	185
5	20/12/2018	50	2.4	219	625	184	30	185
**5**	**03/01/2019**	**52**	**0.3**	**212**	**0**	**0**	**0**	**0**
5	17/01/2019	54	1.0	288	500	147	22.5	139
5	31/01/2019	56	4.2	369	500	147	22.5	139
**5**	**21/02/2019**	**59**	**0.4**	**135**	**0**	**0**	**0**	**0**
5	28/02/2019	60	1.3	289	500	147	22.5	139
6	14/03/2019	62	4.9	299	500	147	22.5	139
6	04/04/2019	65	2.3	332	500	147	22.5	139
6	18/04/2019	67	2.7	313	575	169	22.5	139
6	02/05/2019	69	1.8	339	575	169	25	154
**6**	**16/05/2019**	**71**	**0.5**	**335**	**0**	**0**	**0**	**0**
7	30/05/2019	73	2.9	265	500	147	20	124
7	13/06/2019	75	4.4	213	500	147	22.5	139
7	27/06/2019	77	3.4	252	500	147	22.5	139
7	11/07/2019	79	2.4	329	575	169	22.5	139
7	01/08/2019	82	2.2	341	575	169	22.5	139
7	08/08/2019	83	2.0	341	575	169	25	154
**8**	**29/08/2019**	**86**	**0.4**	**275**	**0**	**0**	**0**	**0**
**8**	**05/09/2019**	**87**	**0.1**	**228**	**0**	**0**	**0**	**0**
8	12/09/2019	88	1.8	320	500	147	22.5	139
8	26/09/2019	90	3.6	269	500	147	22.5	139
8	10/10/2019	92	2.8	232	500	147	22.5	139
8	24/10/2019	94	1.9	236	575	169	22.5	139
8	07/11/2019	96	0.7	310	250	74	20	124

6MP, 6-mercaptopurine; ANC, absolute neutrophil count; MTX, methotrexate; PLT, platelet count

6MP_adj, MTX_adj: dose intensities (%) of 6-mercaptopurine and methotrexate respectively

Supplementary Table S5: ALL maintenance data table of patient UPN_42 highlighting absence of drug titration

ANC, absolute neutrophil count; Hb, hemoglobin; MP, 6-mercaptopurine; MTX, methotrexate; PLT, platelet count; MP_adj and MTX_adj, dose intensities of 6-mercaptopurine and methotrexate respectively (dose intensity, prescribed/ protocol-recommended dose, %); available at the Acute Lymphoblastic Leukaemia maintenance therapy dataset from Mendeley Data ('·' indicates missing data)

**Treatment Cycle**	**Date** dd/mm/yyyy	**Treatment Week**	**ANC** $\times 10^9$/L	**PLT** $\times 10^9$/L	**Hb** g/dL	**MP** mg/week	**MP_adj** %	**MTX** mg/week	**MTX_adj** %
1	03/03/2015	1	0.805	354	10.8	325	92.9	15	90.1
1	17/03/2015	3	1.575	264	11.6	325	92.9	15	90.1
1	31/03/2015	5	1.978	280	10.5	325	92.9	15	90.1
1	28/04/2015	9	0.252	161	11.2	0	0.0	0	0.0
1	05/05/2015	10	0.38	169	11.2	0	0.0	0	0.0
1	12/05/2015	11	1.122	193	11.8	300	85.8	15	90.1
2	26/05/2015	13	3.12	242	12.4	350	100.1	17.5	105.1
2	09/06/2015	15	1.452	223	11.0	350	100.1	17.5	105.1
2	07/07/2015	19	0.64	222	11.1	175	50.0	10	60.0
2	14/07/2015	20	0.992	188	11.1	325	92.9	15	90.1
2	28/07/2015	22	2.275	219	·	325	92.9	15	90.1
2	11/08/2015	24	1.242	247	11.6	325	92.9	15	90.1
3	08/09/2015	28	1.456	185	11.3	325	92.9	15	90.1
3	29/09/2015	31	0.949	135	·	325	92.9	15	90.1
3	27/10/2015	35	1.369	189	·	325	92.9	15	90.1
4	17/11/2015	38	1.44	173	11.8	325	92.9	15	90.1
4	01/12/2015	40	1.08	172	·	325	92.9	15	90.1
4	15/12/2015	42	1.386	260	12.0	325	92.9	15	90.1
4	29/12/2015	44	2.16	175	·	325	92.9	15	90.1
4	19/01/2016	47	1.02	188	·	325	92.9	15	90.1
5	23/02/2016	52	1.54	189	11.6	325	92.9	15	90.1
5	15/03/2016	55	1.44	156	11.8	325	92.9	18	108.1
5	05/04/2016	58	1.4	236	·	325	92.9	18	108.1
6	26/04/2016	61	1.924	249	·	325	92.9	18	108.1
6	10/05/2016	63	2.597	243	·	325	92.9	18	108.1
6	24/05/2016	65	2.494	238	11.8	325	92.9	17.5	105.1
6	07/06/2016	67	1.904	223	11.6	325	92.9	17.5	105.1
6	05/07/2016	71	1	243	·	325	92.9	17.5	105.1
7	02/08/2016	75	1.836	256	10.6	325	92.9	17.5	105.1
7	30/08/2016	79	1.924	192	10.1	325	92.9	17.5	105.1
8	25/10/2016	87	0.912	209	·	325	92.9	17.5	105.1
8	08/11/2016	89	1.248	279	11.1	325	92.9	17.5	105.1
8	29/11/2016	92	5.166	186	11.0	325	92.9	17.5	105.1

Supplementary Table S6: ALL maintenance data table of patient UPN_86 highlighting drug down-titration

ANC, absolute neutrophil count; Hb, hemoglobin; MP, 6-mercaptopurine; MTX, methotrexate; PLT, platelet count; MP_adj and MTX_adj, dose intensities of 6-mercaptopurine and methotrexate respectively (dose intensity, prescribed/ protocol-recommended dose, %); available at the Acute Lymphoblastic Leukaemia maintenance therapy dataset from Mendeley Data ('·' indicates missing data)

Treatment Cycle	Date dd/mm/yyyy	Treatment Week	ANC × 10^9/L	PLT × 10^9/L	Hb g/dL	MP mg/week	MP_adj %	MTX mg/week	MTX_adj %
1	03/03/2016	1	1.81	105	10.6	150	49	7.5	51
1	10/03/2016	2	0.97	86	11.3	150	49	7.5	51
1	17/03/2016	3	2.30	34	10.7	0	0	0	0
1	24/03/2016	4	0.46	15	8.8	0	0	0	0
1	07/04/2016	6	0.10	23	7.8	0	0	0	0
1	21/04/2016	8	1.64	85	8.6	100	33	5	34
1	28/04/2016	9	0.86	113	9.5	100	33	5	34
1	05/05/2016	10	1.50	104	10.2	100	33	5	34
1	12/05/2016	11	2.11	105	11.0	100	33	5	34
1	19/05/2016	12	1.70	81	10.4	100	33	5	34
2	26/05/2016	13	0.48	48	10.3	0	0	0	0
2	02/06/2016	14	0.14	24	9.6	0	0	0	0
2	09/06/2016	15	0.45	34	9.8	0	0	0	0
2	16/06/2016	16	0.56	68	10.6	0	0	0	0
2	23/06/2016	17	1.56	105	10.3	50	16	2.5	17
2	30/06/2016	18	3.31	129	11.1	50	16	2.5	17
2	14/07/2016	20	5.33	105	11.2	50	16	2.5	17
2	21/07/2016	21	1.23	71	11.4	50	16	2.5	17
2	28/07/2016	22	2.30	53	11.1	0	0	0	0
2	04/08/2016	23	0.30	28	10.2	0	0	0	0
3	18/08/2016	25	1.19	94	10.9	50	16	2.5	17
3	25/08/2016	26	1.98	120	11.9	50	16	2.5	17
3	08/09/2016	28	3.30	106	11.7	50	16	2.5	17
3	22/09/2016	30	0.70	57	11.2	25	8	2.5	17
3	06/10/2016	32	0.29	16	8.7	0	0	0	0
3	13/10/2016	33	1.22	65	8.4	0	0	0	0
3	20/10/2016	34	0.78	99	9.4	50	16	0	0
3	27/10/2016	35	0.86	140	10.9	50	16	2.5	17
4	10/11/2016	37	3.54	150	11.3	50	16	0	0
4	08/12/2016	41	0.16	28	10.6	0	0	0	0
4	15/12/2016	42	0.19	20	8.5	0	0	0	0
4	22/12/2016	43	0.37	42	9.4	0	0	0	0
4	29/12/2016	44	0.79	72	9.6	0	0	0	0
4	05/01/2017	45	3.81	128	11.1	25	8	2.5	17
4	12/01/2017	46	4.51	169	·	30	10	2.5	17
4	26/01/2017	48	7.96	154	·	30	10	2.5	17
5	02/02/2017	49	1.63	90	10	15	5	2.5	17
5	16/02/2017	51	0.81	108	·	15	5	2.5	17
5	02/03/2017	53	1.17	72	10.5	15	5	2.5	17
5	16/03/2017	55	2.11	207	·	15	5	2.5	17
5	30/03/2017	57	2.71	80	11.3	15	5	2.5	17
5	20/04/2017	60	1.38	109	·	15	5	2.5	17
6	27/04/2017	61	1.26	101	11.8	15	5	2.5	17
6	11/05/2017	63	2.11	138	·	15	5	2.5	17
6	25/05/2017	65	1.36	102	11.3	15	5	2.5	17
6	08/06/2017	67	3.86	126	·	15	5	2.5	17
6	22/06/2017	69	2.30	105	12.2	15	5	5	34
6	06/07/2017	71	0.87	99	11.9	15	5	5	34
7	20/07/2017	73	1.79	104	·	15	5	5	34
7	03/08/2017	75	1.33	105	11.5	15	5	5	34
7	17/08/2017	77	2.65	126	·	15	5	5	34
7	31/08/2017	79	1.28	158	·	15	5	5	34
7	14/09/2017	81	1.29	99	12.3	15	5	7.5	51
7	28/09/2017	83	1.04	68	·	15	5	5	34
7	05/10/2017	84	2.14	142	·	15	5	5	34
8	19/10/2017	86	2.18	98	·	15	5	5	34
8	26/10/2017	87	0.75	66	10.3	15	5	2.5	17
8	09/11/2017	89	3.23	114	·	10	3	2.5	17
8	16/11/2017	90	0.86	58	9.2	10	3	2.5	17
8	30/11/2017	92	0.31	62	·	0	0	0	0
8	07/12/2017	93	0.81	58	·	0	0	0	0
8	14/12/2017	94	0.34	36	6.7	0	0	0	0
8	21/12/2017	95	0.60	88	10.2	0	0	0	0

Supplementary Table S7 (A and B)

6MP, 6-mercaptopurine; ANC, absolute neutrophil count; AntiMtb, antimetabolite; DI, dose intensity (prescribed / protocol dose); IQR, interquartile range; MT, maintenance treatment; MTX, methotrexate; wm-ANC, weighted mean absolute neutrophil count

Table S7A: ALL Maintenance treatment intensity observations in Cohort 1 (in-person visits alone, n=10) and Cohort 2 (hybrid in-patient and remote monitoring dosing advice, n=10) patients

				Weighted mean dose intensity		
	Patient ID	MT Start Date (dd/mm/yyyy)	wm_ANC ($\times 10^9$/L)	6MP (%)	MTX (%)	AntiMtb (%)
	Sub-Cohort 1: Direct supervision (in-person visits)					
1	UPN_2	05/06/2014	2.00	101	99	107
2	UPN_9	07/01/2014	1.62	89	92	89
3	UPN_12	05/20/2014	1.59	70	67	53
4	UPN_18	09/08/2014	1.75	96	94	97
5	UPN_40	01/13/2015	1.78	91	94	90
6	UPN_64	08/13/2015	1.89	48	59	36
7	UPN_68	07/07/2015	1.55	85	66	63
8	UPN_78	11/16/2015	1.63	59	75	52
9	UPN_86	03/03/2016	1.74	8	18	2
10	UPN_127	09/08/2016	1.90	90	80	80
	Sub-Cohort 2: Hybrid supervision (in-person and remote supervision over e-mail					
1	UPN_359	06/18/2020	2.26	57	53	35
2	UPN_378	07/09/2020	1.59	46	75	39
3	UPN_384	07/23/2020	2.04	92	84	85
4	UPN_391	10/29/2020	1.29	112	110	137
5	UPN_395	09/03/2020	1.93	92	98	111
6	UPN_397	10/08/2020	3.67	71	108	80
7	UPN_400	10/29/2020	1.43	61	55	37
8	UPN_411	12/31/2020	1.95	78	73	65
9	UPN_436	02/18/2021	1.92	85	91	89
10	UPN_445	04/22/2021	1.39	76	125	101

wm_ANC, weighted mean absolute neutrophil count

6MP, 6-mercaptopurine; MTX, oral methotrexate; AntiMtb, antimetabolite

Table S7B: Comparison of weighted mean neutrophil counts and drug intensities during ALL maintenance between patient sub-cohorts 1 and 2

Parameters		Sub-Cohort 1	Sub-Cohort 2	p-value*
ANC	Median	1.75	1.94	0.53
($\times 10^9$/L)	IQR	1.62 - 1.86	1.47 - 2.20	
6MP-DI	Median	87	77	0.91
(%)	IQR	62-91	63 - 90	
MTX-DI	Median	77	88	0.44
(%)	IQR	66-93	73-106	
AntiMtb-DI	Median	72	83	0.68
(%)	IQR	52-90	46-98	

*Mann-Whitney
