# Supplementary material for: Analysis toolkit for evaluation of drug titration practice in acute lymphoblastic leukemia maintenance
Source: JAMIA Open. 2024 Sep 13;7(3):ooae089. doi: 10.1093/jamiaopen/ooae089 (PMC11398893; doi:10.1093/jamiaopen/ooae089)
Supplement: ooae089_Supplementary_Data [file ooae089_supplementary_data.zip › Supplementary_Figure_S1.pdf]

## ALL Maintenance: Visualising drug titration practice in individual patients

Longitudinal tracking of neutrophil counts and antimetabolite drug doses through ALL maintenance

| (A) At each dosing visit |
| --- |
| Neutrophil count (ANC) |
| Doses of 6MP and MTX |

ANC, absolute neutrophil count
6MP, 6-mercaptopurine (mg/week)
MTX, methotrexate (mg/week)

| (B) For each ALL MT cycle |
| --- |
| Weighted mean ANC |
| Weighted mean AMtb-DI |

MT, maintenance treatment
ANC, absolute neutrophil count
AMtb-DI, antimetabolite dose intensity

### Recurring Drug Titration Patterns

| Observed Patterns | Interpretation | Probable explanation(s) |
| --- | --- | --- |
| **Titration to Tolerance** | Highest tolerated drug doses | Recommended practice |
| **Serial Drug Up-Titration** | Tolerated doses not established | Likely: Patient non-adherence[a]<br>Sometimes: Skewed drug metabolism[b] |
| **Serial Drug Down-Titration** | Prescribed below-protocol doses | Likely: Poor drug metaboliser[c]<br>Also: Non-haem clinical toxicity[d]<br>Often: Conservative prescribing[e]<br>Sometimes: Other haem toxicity[f] |
| **No Drug Titration** | Fixed doses through treatment | Prescriber non-compliance |
| **Mixed Titration Patterns** | Combinations of titration patterns | Temporal variations in dosing variables[g] |

Dose intensity, prescribed dose as a proportion of protocol-recommended dose

Antimetabolite dose intensity, product of dose intensities of 6-mercaptopurine and methotrexate

[a]Patient non-adherence and persistent high blood counts, explaining serial drug dose escalation

[b]Lower intracellular thioguanine nucleotide levels from methylation shunting of 6MP

[c]Constitutional polymorphisms in genes encoding drug metabolising enzymes

[d]Including severe viral infections or toxicities arising from excess methylated 6MP metabolites

[e]Hesitation to escalate doses, especially in patients with haematological and/or clinical toxicities

[f]Isolated low platelet count or low haemoglobin, due to disease non-remission or unusual drug toxicities

[g]Temporal variations in factors that influence dose titration decisions, such as clinical toxicities

**Figure S1:** Characteristic line plot patterns of antimetabolite dose titration practice (at each dosing visit and for each 12-week cycle) in individual patients during the ALL maintenance treatment phase, with accompanying probable explanations for the observed dose titration patterns
